# Modification of the Persian version of Hermans Achievement Motivation Questionnaire to develop an adapted scale for measuring motivation of post-stroke survivors in Iran

**Published:** 2016-10-07

**Authors:** Seyed Alireza Derakhshanrad, Emily Piven

**Affiliations:** 1 Department of Occupational Therapy, School of Rehabilitation Sciences AND Rehabilitation Sciences Research Center, Shiraz University of Medical Sciences, Shiraz, Iran; 2 University of Texas at El Paso, El Paso, Texas AND Health Matters First of Florida, Inc., Oakland, FL, USA

**Keywords:** Rehabilitation, Motivation, Cerebrovascular Accident

## Abstract

**Background:** Research has shown that in order for recovery from a stroke to occur, motivation for recovery has been essential component of rehabilitation. Researchers and clinicians have tended to categorize stroke survivors subjectively into two groups: those who have been motivated or unmotivated, perhaps due to the paucity of objective measures that distinguish the groups. Since classification of clients based on subjective inference would be prone to bias, this pilot study aimed to establish a regionally validated scale that was adequately standardized for measuring motivation of adult post-stroke survivors in Iran.

**Methods:** The Persian version of Hermans Achievement Motivation Questionnaire (PHAMQ) was identified as the best test for the purposes of this study. A multistep process was undertaken to create an adapted scale from the PHAMQ that focused on functional behaviors, often seen in the process of rehabilitation. Thus, the Adapted Achievement Motivation Questionnaire (AAMQ) was examined for reliability and validity. Cronbach’s alpha was used for measuring internal consistency and expert panel opinions were sought to analyze the content validity of AAMQ.

**Results:** A convenience sample of 25 stroke subjects comprised of 10 males and 15 females participated in this study with the mean age [(± standard deviation (SD)] 58.3 ± 9.8 years and range of 35-72 years. Expert opinion regarding the relevance of AAMQ items led to provide compelling evidence for a 28-item AAMQ. Cronbach’s alpha of 0.946 showed a perfect internal consistency for test items.

**Conclusion:** This pilot study suggested that AAMQ could be utilized as a regionally validated scale for examining the motivational level of patients who have sustained strokes in Iran. Further research are recommended.

## Introduction

Cerebrovascular accidents referred to as strokes, have been a common healthcare problem globally.^1^ The highest incidence of disabilities that affect everyday activities have been reported among stroke survivors (SS) compared to other common long term health problems such as musculoskeletal disorders and heart conditions.^2^ Rehabilitation, a multifaceted time-consuming process, has included behavioral goal-setting and therapeutic intervention aimed to reduce the disability and improve function of SS.^3^ Rehabilitation practitioners strive to find validation of outcomes in evidence-based practice^4^ for reimbursement. However, the outcome of rehabilitation interventions is depended on how much the client demonstrates that he/she wants to recover. Accordingly, if the client expresses desire to pursue rehabilitation goals, he/she would be considered a motivated client.^5^ One correlational study with 200 subjects quantitatively determined that motivation towards treatment played a role in achieving favorable outcomes and predicted the likely functional outcomes of SS.^6^

The World Health Organization described motivation as “a driving force for action” and an essential element of mental function (p.51).^7^ While being embedded in the rehabilitation realm, motivation provided a “framework by which rehabilitation professionals and their clients can work together to enhance the client’s physical independence and psychological well-being” (p.7).^8^ This client-practitioner framework averted clients becoming disappointed while participating in rehabilitation process.^9^

There has been consensus among researchers and practitioners that people who have had strokes fit subjectively into motivated and unmotivated categories.^6^^,^^10^^,^^11^ Categorizing motivation as simply black or white has suggested the bias of subjectivity, perhaps due to the paucity of objective measures. There were only two quantitative studies suggesting some questionnaires that could be utilized for measuring the motivational level of stroke survivors.^12^^,^^13^

Hallams and Baker warned the professionals that subjectively assessing the clients’ motivation could result in unreal inference about the construct of motivation.^12^ Using search words for both motivation and rehabilitation, they developed a two-part questionnaire with 30 items to assess motivation in Australian subjects with strokes. Three experts and three clients with strokes participated in the study to give their opinions, in order for the researchers to determine the content validity of the questionnaire. All the items that were included in the questionnaire were scored a Cronbach’s value greater than 0.9, showing that all the items measured the construct of motivation in the field of rehabilitation.

Emphasizing the necessity of developing an instrument for objectively measuring the motivation of subjects with strokes, White et al.^13^ devised a 28-item assessment tool, which was adapted from the Sports Motivation Scale (SMS) and re-named the Stroke Rehabilitation Motivation Scale (SRMS). The SRMS, comprised of 7 sets of questions, was tested on 31 Australian participants with acute post-stroke conditions. Based on standardization statistical results such as intra-class correlation coefficients, Cronbach’s alpha, and item-to-total correlation coefficients, a final 7-item SRMS was proved to have good reliability among the rest of 28 items, but validity of the questionnaire was not established.^13^

The authors of this paper found no other validated instruments to measure motivation of clients who have had strokes. This gap in the literature justified the specific aim of this study, which was to establish a regionally validated motivational scale that was adequately standardized to measure the motivational level of Iranian SS towards rehabilitation. Thus, our primary objective in this paper was to conduct preliminary research with a number of expert panelists and a sample of SS to provide a Farsi version of a motivation questionnaire, the findings of which could be utilized for future investigations so that the rehabilitation clinical care might be improved by using such questionnaire.

## Materials and Methods

To this end, the Hermans Achievement Motivation Questionnaire (HAMQ),^14^ was identified as the best test for the purposes of this pilot study because the Persian version of this scale had been developed through forward and backward translation of the scale into Persian with questions adapted to the Iranian culture, and then validated for an Iranian population of students.^15^

The Persian version of Hermans Achievement Motivation Questionnaire (PHAMQ), was chosen for present study for two reasons. Firstly, because this scale has been included a number of different behavioral domains under one construct, the “achievement motive”.^14^ Secondly, this scale had been widely used in studies in Iran. The questionnaire contained 29 incomplete statements along with four-choice items for each statement, whereby the responder was allowed to choose one to complete the incomplete sentence. The item choices were scored on a four-point numerical Likert scale from 1 to 4. The scale was used to screen individuals quantitatively for different motivational levels. The scale covered 10 aspects of behavior in order to measure the “achievement-oriented” situation of students when they express specific patterns of academic demeanor such as: being achievement-motivated, persistent, and diligent in doing their academic tasks. The rich quality of the initial item pool with 92 items was the chief importance in construction of this scale.^14^ PHAMQ was standardized on 1073 (560 females and 513 males) high school students of city of Saveh, Iran. The test was confirmed to have high reliability with a Cronbach’s alpha of 0.803. The validity of the test was also confirmed by performing the construct validity procedure.^15^

Creation of a modified scale from PHAMQ was a four step process, in order to assess the same construct for Iranian adults who had experienced strokes. Firstly, permission was granted for the first author to adapt the HAMQ from its originator. Secondly, the PHAMQ underwent several purposive amendments by the first author, who substituted its academic-oriented performance orientation for a therapeutic task-oriented performance orientation, in order to change the construct to have a rehabilitation focus. Table 1 below shows several examples of some amended items and their corresponding items in PHAMQ. A full version of the adapted scale in Farsi may be obtained from the first author.

Thirdly, a five-person expert panel, comprised of three experienced occupational therapists and two physiotherapists who worked in rehabilitation settings, was created to review the adapted questionnaire for its suitability for stroke survivors. The content validation procedure led to removal of one item, resulting in a new questionnaire with 28 items. The new questionnaire, named the Adapted Achievement Motivation Questionnaire (AAMQ), has a total score of 28 to 112, whereby the higher scores indicated the higher achievement motivation.

The fourth step was to design an observational descriptive study, so that the reliability of the AAMQ could be examined by the means of calculating Cronbach’s alpha coefficient for investigating internal consistency. An internal consistency is one type of reliability that determines whether test items of a questionnaire all measure the same behavior or construct. Coefficients higher than 0.7 are considered to be optimal.^16^

**Table 1:** Examples of amended items and their correspondence

**Amended items for the AAMQ**	**Corresponding items from PHAMQ**
“While performing therapeutic exercises, I think perseverance is ______.”	“At university, I think perseverance is ______.”
Very unimportant	Very unimportant
Rather unimportant	Rather unimportant
Important	Important
Very important	Very important
“My physicians and therapists think I am ______.”	“At university, they think I am ______.”
Very diligent	Very diligent
Diligent	Diligent
Rather easy-going	Rather easy-going
Very easy-going	Very easy-going
“Good relations with my physicians and therapists ______.”	“Good relations with my teachers at university ______.”
Are appreciated very much	Are appreciated very much
Are appreciated	Are appreciated
Are not to be so important	Are not to be so important
Are completely unimportant	Are completely unimportant
“I found patients who make more effort are ______.”	“At university I found classmates who study very hard are ______.”
Very successful	Very nice
Successful	Nice
Not successful	Not nice
Not successful at all	Not nice at all

AAMQ: Adapted Achievement Motivation Questionnaire; PHAMQ: Persian version of Hermans Achievement Motivation Questionnaire

The research project was approved by the Research and Ethics Committee of Shiraz University of Medical Sciences, Iran. Separate consent forms were obtained from the five rehabilitation professionals and participants who had suffered strokes and met the inclusion criteria: willingness to participate, 18-75 years old, sub-acute ictus between 3 to 12 months post-stroke, and presentation of sufficient cognitive and communicative capability to participate in the study. Participants were recruited through a convenience sampling from four rehabilitation centers, which were well-known for offering rehabilitation services to stroke subjects. Molazade et al.^17^ study provided sample size guidelines, to calculate the minimum required sample size of 25 subjects, using the sample size equation N=(Z1-α2+Z1-β)2S2d2, where α = 0.05, β = 0.2, d = 4, and mean ± SD of PHAMQ for their population study were 72.8 ± 7.2.

## Results

The content validity of the 28-item AAMQ was approved by an expert panel. A convenience sample of 25 subjects who sustained strokes and included 10 males and 15 females was enrolled in this study. The sample mean age was 58.3 ± 9.8 years. The minimum and maximum age of participants was 35 and 72 years, respectively. From the age-related point of view, there was no statistically significant difference between males (59.6 ± 10.2 years) and females (57.4 ± 9.8 years).

The total scale mean was 74.3 (SD 8.1, range 57–94). There was no statistically significant difference between the total score of males (74 ± 6.7) and females (74.5 ± 9.2). Cronbach’s alpha of 0.946 showed a perfect internal consistency for test items indicated that all items measure the same construct. Table 2 below shows item-to total correlation that indicated the acceptable correlation between each test item and the total test score. All items seemed to be worthy of retention because they all correlated with the total scale to a good degree (the lowest r = 0.3).

## Discussion

The present pilot study examined the statistical psychometric qualities of the AAMQ using a small sample of Iranian subjects who were post-stroke. The content validity and internal consistency demonstrated perfect values. Despite its preliminary character, the research reported here would seem to indicate that AAMQ could be used with some confidence to screen the achievement motivation trait of SS residing in Iran. 

**Table 2:** Item to total correlation between test items and total test score

**Item number** **(Question)**	**Item-total ** **correlation**	**Cronbach's alpha ** **if item deleted**
1	0.83	0.941
2	0.81	0.941
3	0.83	0.941
4	0.51	0.945
5	0.61	0.944
6	0.85	0.941
7	0.30	0.947
8	0.54	0.944
9	0.87	0.940
10	0.44	0.945
11	0.34	0.946
12	0.76	0.942
13	0.64	0.944
14	0.43	0.945
15	0.45	0.945
16	0.56	0.945
17	0.78	0.942
18	0.69	0.944
19	0.82	0.942
20	0.58	0.944
21	0.43	0.945
22	0.36	0.946
23	0.31	0.946
24	0.62	0.944
25	0.51	0.945
26	0.74	0.943
27	0.44	0.945
28	0.77	0.942

Maclean and Pound^10^ argued that motivation was a highly subjective concept with a lack of clinical consensus regarding its definition. They alluded to some sort of “acceptable modes of patient behavior in rehabilitation” (p.497), as an indication of the inferences made by the rehabilitation professionals about the demeanor of clients, with regard to their willingness or unwillingness to participate in rehabilitation interventions.^10^ Having considered the disadvantages of subjectively categorizing the stroke survivors’ motivation into high and low level terms, it can be argued that the scale developed in this study provided preliminary supportive evidence for quantitatively classifying subjects with strokes by levels on a motivation continuum.

Following the three-part validity assessments, according to the ‘trinitarian’ model of validity, content validity was only considered in this study. The other two parts are criterion-related validity and construct validity which lead to gathering further supportive evidence about the validity of a test.^18^

Comparing the scores obtained from an instrument with the scores of a measure of interest, which is called criterion or gold-standard, is the method of determining the criterion-related validity of an instrument.^19^ It was not possible to determine criterion validity of AAMQ because there was no regionally standardized measure for comparison at the time of the study. However, one suggestion for future studies would be to design correlational research in order to evaluate the statistical relationships between the AAMQ and other scales, such as Connor-Davidson Resilience Scale (CR-RISC), which was standardized on a sample of Iranian subjects who had experienced strokes.^20^ CR-RISC was also originally reported to measure achievement motivation as its latent construct factor in a sample of Iranian students.^21^

Factorial analysis, one of the most frequently used statistical strategy for evaluating the construct validity,^16^ was also impossible to perform in the present study due to the small sample size. However, as Hoomon and Asgari argued, there were 7 factors that described the latent construct of the PHAMQ.^15^ The factors were perseverance, self-esteem, time-perception, seeking opportunities, diligence, competency, high ambition, and foresight. There is, perhaps, some truth in the idea of yielding the same factors from the adapted scale standardized in the present study.

This pilot study addressed the need to develop a Farsi version of a motivation questionnaire and examined whether the questionnaire had a minimum acceptable level of validity and reliability. This study allowed the authors to examine the content validity and internal consistency (reliability) of the AAMQ. Further research should be accomplished with a larger sample population to determine the other types of validity and reliability. In addition, conducting research on a larger sample size for statistical comparison to other rehabilitation outcome measure questionnaires would be required to determine the usefulness or clinical utility of AAMQ in classifying the subjects into different kinds of motivational groups.

## Conclusion

The findings of this pilot study were promising. The AAMQ could be utilized by researchers and clinicians as an objective measure to examine the motivational level of Iranian patients who have experienced strokes. Rehabilitation professionals could use the tool to classify levels of motivation of clients and recognize need for re-motivation treatment early in the process of recovery. Further, the capacity to quantify motivational levels has implications for identifying those patients that may improve faster with early treatment of post-stroke depression. Having a tool that objectively measures change in levels of motivation can also provide opportunity for Iranian clinicians to measure outcomes of interventions that provide evidence for practice.

## Notes:

**How to cite this article:** Derakhshanrad SA, Piven E. Modification of the Persian version of Hermans Achievement Motivation Questionnaire to develop an adapted scale for measuring motivation of post-stroke survivors in Iran. Iran J Neurol 2016; 15(4): 189-94.
